# Anti-carcinoembryonic antigen-related cell adhesion molecule antibody for fluorescence visualization of primary colon cancer and metastases in patient-derived orthotopic xenograft mouse models

**DOI:** 10.18632/oncotarget.27446

**Published:** 2020-01-28

**Authors:** Hannah M. Hollandsworth, Siamak Amirfakhri, Filemoni Filemoni, Verena Schmitt, Gunther Wennemuth, Alexej Schmidt, Robert M. Hoffman, Bernhard B. Singer, Michael Bouvet

**Affiliations:** ^1^Department of Surgery, University of California, La Jolla, CA, USA; ^2^Moores Cancer Center, University of California San Diego, La Jolla, CA, USA; ^3^Institute of Anatomy, University Hospital, University Duisburg-Essen, Essen, Germany; ^4^AntiCancer, Inc., San Diego, CA, USA; ^5^VA San Diego Healthcare System, San Diego, CA, USA; ^6^Department of Medical Biosciences, Pathology, Umeå University, Umeå, Sweden; ^*^These authors contributed equally to this work

**Keywords:** carcinoembryonic antigen, CEACAM, colon cancer, fluorescence, near-infrared

## Abstract

Background: Monoclonal antibody (mAb) 6G5j is a novel anti-CEACAM monoclonal antibody. Our aim was to investigate mAb 6G5j binding characteristics and to validate fluorescence targeting of colorectal tumors and metastases in patient derived orthotopic xenograft (PDOX) models with fluorescently labeled 6G5j.

Materials/Methods: The MAb 6G5j binding profile was analyzed with ELISA, Western blot and immunohistochemistry. MAb 6G5j was conjugated to near-infrared dye IR800CW (LI-COR). Western blotting was performed with various colon cancer cell lysates to determine CEACAM expression. Nude mice received orthotopic implantation of patient-derived primary colon cancer and patient-derived colon cancer metastases. Mice were administered varying doses of 6G5j-IR800CW via tail vein injection and imaged 24 and 48 hours later.

Results: MAb 6G5j bound to human CEACAM1, 3, 5, 6 and 8. Western blotting demonstrated varied expression of CEACAMs in 15 of 16 colon cancer lysates. Dose and time-response imaging demonstrated optimal imaging 48 hours after administration of 50 μg 6G5j-IR800CW (Tumor-to-liver ratio (TLR) 3.17, SEM ± 0.45). Primary cancers and multiple metastases were fluorescently visualized.

Conclusions: Anti-CEACAM antibody 6G5j binds multiple CEACAMs which may lead to improved detection of tumor margins for tumors and metastases that have variable expression of CEA and other CEACAMs. 6G5j mAb may be useful for colon cancer detection for pre-surgical diagnosis and fluorescence-guided surgery.

## INTRODUCTION

Early diagnosis and surgical resection of a wide variety of epithelial malignancies remain a vital challenge due to difficulty of intraoperative recognition of tumor margins and small metastases during minimally invasive procedures. There is also difficulty in achieving negative margins in colorectal cancer due to the relatively small surgical field afforded by the pelvis. Positive margin rates of colorectal cancer surgery have been previously reported at 6.83% overall, which contribute to local recurrence and metastases [1]. Improved visualization during minimally invasive procedures may assist in the reduction of positive margins and local recurrence. In addition, otherwise invisible small metastases may also be visualized with improved imaging techniques.

Carcinoembryonic antigen-related cell adhesion molecules (CEACAMs) are members of the carcinoembryonic antigen (CEA) gene family and the immunoglobulin (Ig) superfamily, consisting of 12 immunoglobulin-related cell surface glycoproteins. These molecules play a role in cell signaling, cell adhesion and tumorigenesis [2, 3]. CEACAMs are described as multifunctional glycoproteins with a distinct expression in certain epithelial, endothelial and immune cells [4]. While immune cells and endothelium solely express CEACAM1, human epithelial cells show a much more complex CEACAM expression pattern. CEACAM1, CEACAM5 and CEACAM6 are co-expressed in epithelial cells of the gastro-intestinal tract and can also be over-expressed in endometrial, lung, ovarian, cervical, breast and colon cancers [5]. CEACAMs participate in multiple physiological and pathophysiological processes, including cell-cell adhesion, epithelial differentiation, apoptosis, neo-vascularization and pro-inflammatory regulation of B- and T-cell proliferation [4, 6, 7]. Alterations in the expression of CEACAM are often linked to epithelial disorders and malignancies. CEACAM1 has been implicated in the progression of colon cancer and is considered a biomarker of colorectal cancer [8]. The human CEA (also named CEACAM5) and CEACAM6 have also been shown to be strongly positive in most colon cancers [8, 9].

Prior studies have successfully utilized fluorescently-labeled antibodies in mouse models to specifically visualize pancreatic and colorectal tumors [10–12]. One preclinical study demonstrated that fluorophore-conjugated anti-CEA antibodies specifically targeted CEA-expressing tumors in patient-derived orthotopic xenografts (PDOX) and orthotopic cancer cell-line mouse models [12]. While anti-CEA antibodies conjugated to fluorophores have enabled tumor visualization, the simultaneous targeting of multiple antigens may further enhance visualization, provide more distinct tumor margins and improve detection of metastases. In this study, we test a novel anti-CEACAM antibody (6G5j) conjugated to a fluorescent dye for detection of multiple CEACAM antigens to enhance visualization of colorectal PDOX tumors and metastases in murine models.

## RESULTS

**Figure 1:**
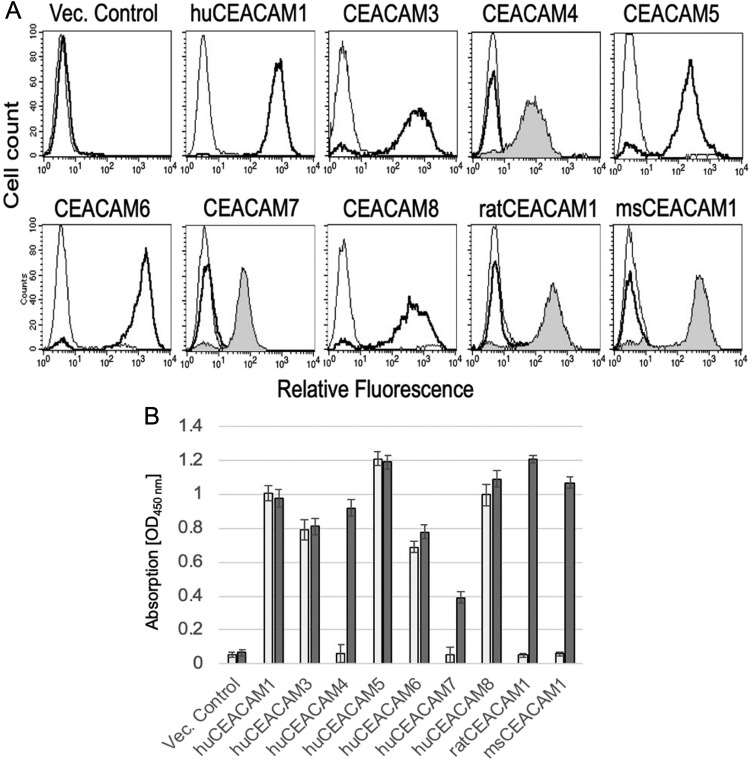
(**A**) Flow cytometric analyzes of mAb 6G5j with CHO transfectants expressing human, rat and mouse CEACAMs. MAb 6G5j binding is shown as thick line, isotype matched IgG thin line and positive control staining gray-filled histogram. In panel (**B**), the results in (A) were confirmed by ELISA in which indicated lysates were immobilized and detected by mAb 6G5j (white bars) or positive control staining (grey bars) followed by incubation with HRP-coupled secondary antibody and the chromagen substrate TMB. Data are representative of three independent experiments.

**Figure 2:**
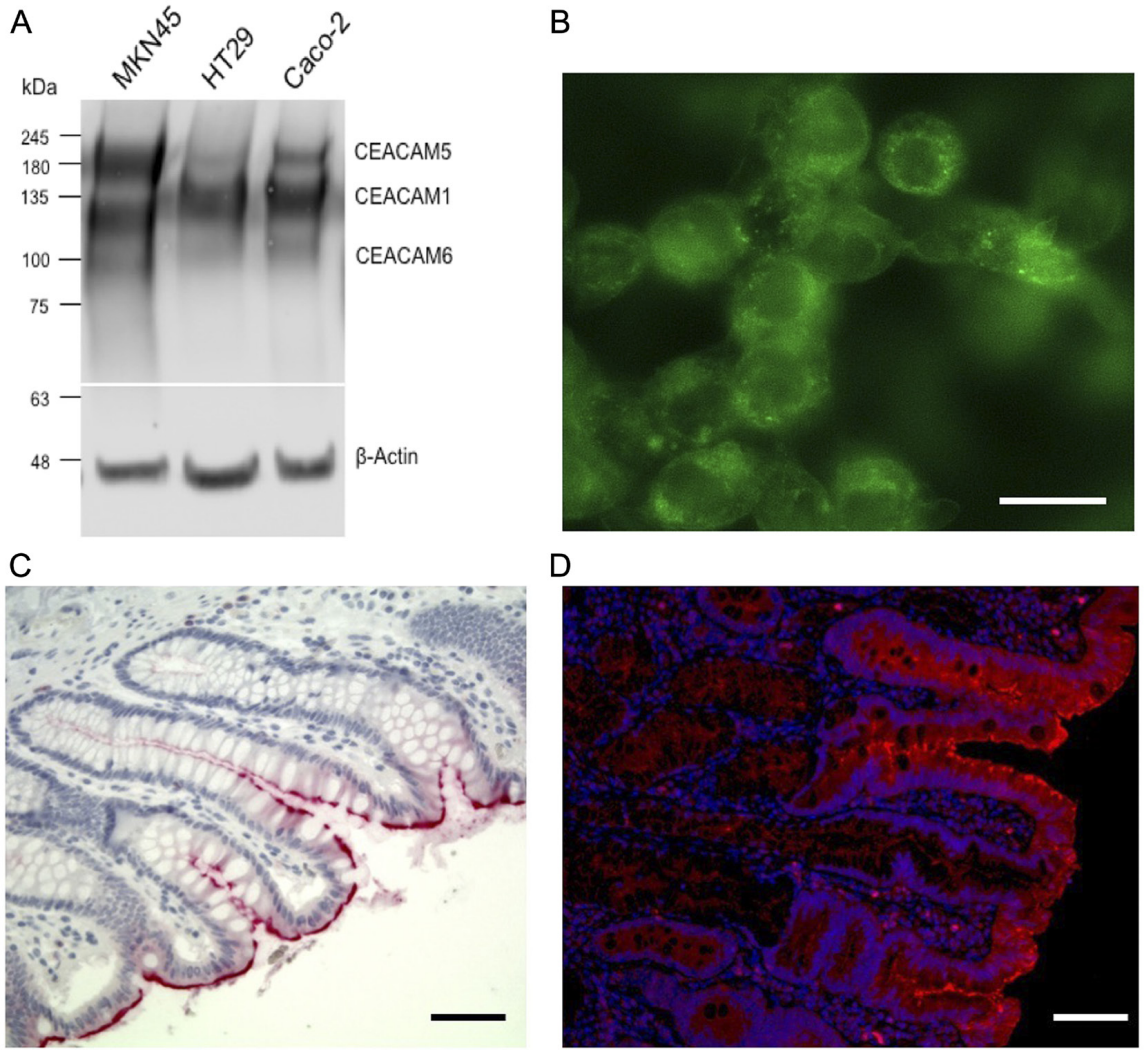
Colon cancer cell line western blot, fluorescence staining and immunohistochemistry of CEACAM expression. (**A**) Western blot recognition pattern of mAb 6G5j in lysates of gastric (MKN45) and colon (HT29, Caco-2) cancer derived cell lines. (**B**) Fluorescence staining of paraffin-embedded HT29 human colon cancer cells labeled with 10 µg/ml mAb 6G5J and detected by Alexa 488 coupled secondary anti mouse Fab2 antibody. (**C**) Immunohistochemistry of paraffin-embedded human colon and (**D**) jejunul tissues incubated with 10 µg/ml mAb 6G5J. Scale bar in panel b represents 10 µm. Scale bars in panels c and d represent 50 µm. Data are representative of two independent experiments.

Flow cytometry utilizing transfectants expressing human, rat and mouse CEACAM1, CEACAM3, CEACAM4, CEACAM5, CEACAM6, CEACAM7 and CEACAM8 as well as rat and mouse CEACAM1 demonstrated that mAb 6G5j binds human CEACAM1, CEACAM3, CEACAM5, CEACAM6 and CEACAM8 (Figure 1A) but not CEACAM1 of mouse and rat origin. Isotype matched IgG and antibodies binding CEACAM4, CEACAM7, rat CEACAM1 and mouse CEACAM1 were applied for negative and positive control staining, respectively. Samples were analyzed by flow cytometry and results were confirmed by ELISA. Indicated lysates were immobilized and detected by mAb 6G5j or positive control staining antibodies (Figure 1B). By western blotting we found that mAb 6G5j detected endogenously expressed CEACAM1, CEACAM5 and CEACAM6 in lysates of epithelial cell lines derived from gastric (MKN45) and colon (HT29, Caco-2) cancer cell lines (Figure 2A). The fraction of CEACAM1, CEACAM5 and CEACAM6 varies in the different cell lines. Next, we analyzed the performance of mAb 6G5j in immunohistochemical staining utilizing paraffin-embedded HT29 cells (Figure 2B) and sections of colon (Figure 2C) and jejunum (Figure 2D), and found a binding profile that corresponded to what was previously found in the literature [13, 14].

**Figure 3:**
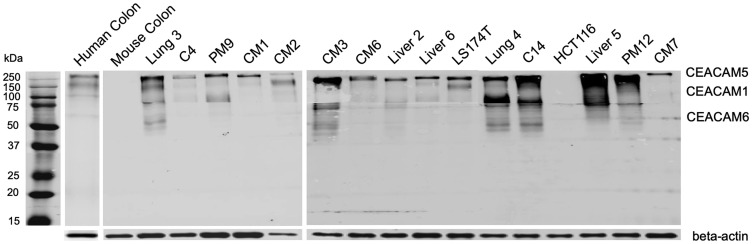
Whole membrane western blot of 6G5j-IR800CW for CEACAM expression in colon cancer lysates. LS174T and HCT116 represent human colon cancer cell-lines. Lung 3, C4, PM9, CM1, CM2, CM3, CM6, Liver 2, Liver 6, Lung 4, C14, Liver 5, PM12 and CM7 represent patient-derived colon cancer surgically obtained from our institution (UCSD). The expected molecular weights for CEACAM1, CEACAM5 and CEACAM6 are approximately 120–150 kDa, > 200 kDA and 60–90 kDa respectively. Beta-actin was used as a control. The three separate membranes shown are independent results.

Further western blotting of mAb 6G5j conjugated to IR800CW demonstrated strong expression of CEACAM5 in 15 of 16 colon cancer lysates (Figure 3). Normal human colon expressed small amounts CEACAM 1, 5 and 6 (Figure 3). Mouse colon and human colon cancer cell line HCT116 did not express CEACAMs (Figure 3). Human colon cancer cell line LS174T, patient colon cancer lung metastases (lung 3, lung 4), regional and liver metastases (CM1, CM2, CM3, CM6, CM7, liver 2, liver 6, liver 5), peritoneal metastases (PM9, PM12) and primary colon cancer (C4 and C14) all demonstrated varying levels of CEACAM5. Colon cancer patient lung 3, PM9, CM3, lung 4, C14, liver 5 and PM12 also expressed CEACAM1 and 6 (Figure 3). The differences in apparent molecular weight of CEACAM expression between different lysates in Figure 3 can be explained by the variable glycosylation sites in the domains of CEACAM molecules [3].

**Figure 4:**
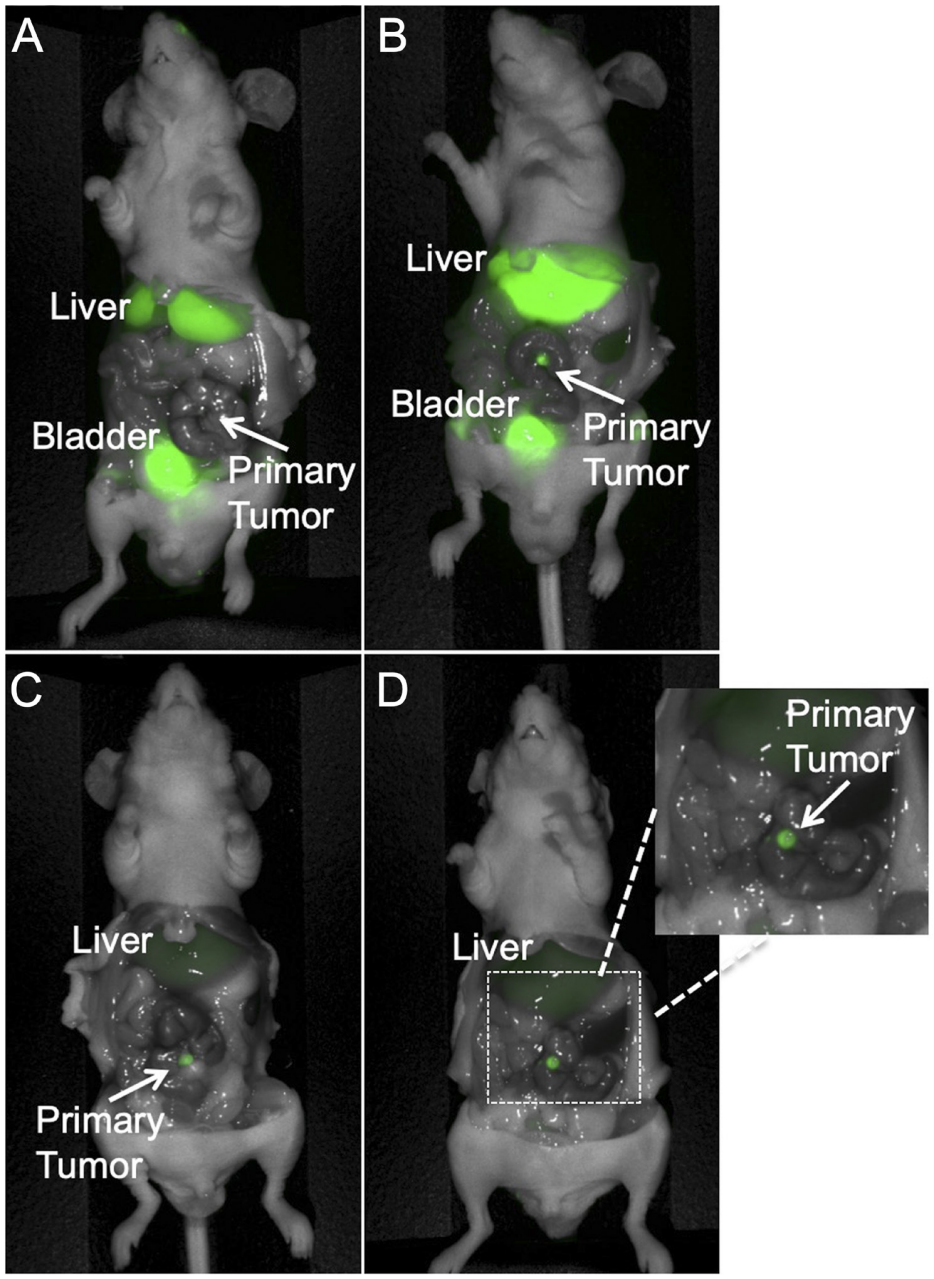
Representative dose response imaging of 6G5j-IR800CW in a PDOX model established with tumor implantation to the colon with patient colon cancer metastasis to the lung (Lung 4). (**A**) The mouse received 25 mcg 6G5j-IR800CW and was imaged after 24 hours. TLR = 0.394. (**B**) The mouse received 50 mcg 6G5j-IR800CW and the image was obtained 24 hours after administration. TLR = 0.638. Fluorescence of the bladder in (A) and (B) is due to excretion of IR800CW dye in urine. (**C**) The mouse received 25 mcg 6G5j-IR800CW and the image was obtained 48 hours after administration. TLR = 2.192. (**D**) The mouse was imaged 48 hours after administration of 50 mcg 6G5j-IR800CW, TLR = 2.637.

**Figure 5:**
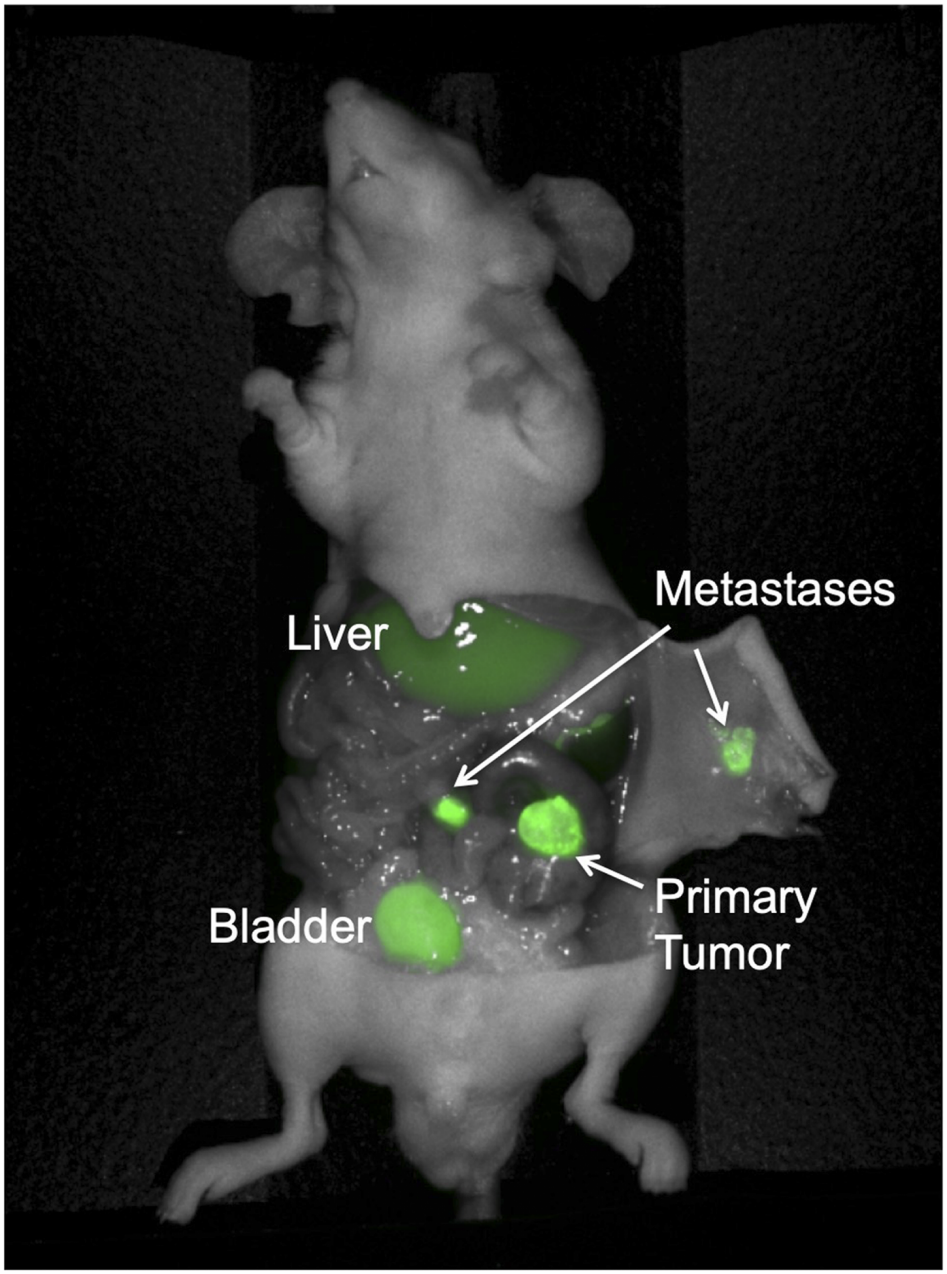
Colon cancer PDOX model with regional metastases, implanted on the cecum with patient-derived primary colon tumor sample Lung 4. The mouse was administered 50 mcg 6G5j-IR800CW and imaged 48 hours after administration. Fluorescence of the bladder is due to excretion of IR800CW dye in urine.

Dose and time-response imaging demonstrated that the tumor-to-liver ratio (TLR) 24 hours after administration of 6G5j-IR800CW was less than 1, presumably due to metabolism of the dye in the liver and relatively low sequestration of the antibody-fluorophore conjugate in the tumor at this time point (Figure 4). The TLR increased 48 hours after tail vein administration, with a TLR of 2.637 after administration of 50 μg of 6G5j-IR800CW (Figure 4). This is likely due to clearance of the dye metabolites from the liver and enhanced sequestration of antibody-fluorophore conjugate in the tumor over time. Additional PDOX models of C4 (*n* = 1) and lung 4 (*n* = 4) were imaged 48 hours after administration of 50 μg of 6G5j-IR800CW with a mean TLR of 3.17 (SEM ± 0.45). The TLR of the C4 PDOX model, which demonstrated a lower expression of CEACAMs on Western blot compared to lung 4, was 2.64, while the mean TLR for lung 4 tumors alone was 3.27 (SEM ± 0.54) (Figure 3). As seen in Figure 5, one PDOX model (lung 4) developed regional metastases. The Lung 4 PDOX model was imaged 48 hours after administration of 50 μg 6G5j-IR800CW, which enabled visualization of clear primary tumor margins as well as multiple intra-abdominal metastases that spontaneously developed (Figure 5). Non-invasive imaging of a control mouse injected with a non-specific antibody conjugated to IR800CW demonstrated fluorescence of the entire mouse without any sequestration in a subcutaneous patient-derived tumor.

After mice were euthanized and imaging was performed, internal organs were removed and examined to determine if gross toxicity was present. There were no gross defects of internal organs to suggest toxicity. No mice had to be euthanized preemptively during the study due to toxicity or adverse effects.

## DISCUSSION

The results of the present study suggest that anti-CEACAM antibody 6G5j is effective for fluorescence targeting and imaging of colon cancer. CEACAM1, CEACAM5 and CEACAM6 have been demonstrated to be over-expressed in certain epithelial cancers, such as colon cancer [4]. However, it seems to be variable whether all or just some of the three CEACAM members become up-regulated in individual tumors, as seen in Figure 2A. Therefore, targeting of multiple CEACAMs may improve colon cancer detection if variable expression of the different CEACAMs exists within a tumor. In this study, we identified that mAb 6G5j is able to bind to CEACAM1, CEACAM3, CEACAM5, CEACAM6, and CEACAM8 and thus could be a valuable tool to identify malignant epithelial cells in which at least one of the CEACAMs is over-expressed. In addition, 6G5j-IR800CW labels colon cancer metastases, which can aid in intra-operative detection of small metastases invisible on pre-operative imaging. The optimal timing of imaging was 48 hours after intravenous administration of 50 μg of fluorescently-labeled 6G5j, with an overall mean TLR of 3.17. Lung 4 PDOX models, which demonstrated higher expression pattern of CEACAMs on Western blot than C4 (Figure 3), had a higher mean TLR (3.27) compared to the TLR of a C4 PDOX mouse model imaged (TLR = 2.64).

Fluorescence-guided surgery continues to be an emerging field for surgical oncology. In general, few studies have been performed to determine successful antibodies for specific visualization of primary and metastatic colorectal cancer [9, 15]. In the present study, we demonstrate that multiple CEACAMs, which are specifically over-expressed in colorectal cancers [8, 9, 16], are a useful target for fluorescence imaging of primary and metastatic colorectal tumors. In prior mouse studies, it has been demonstrated that CEA antibodies are useful for fluorescence imaging of colon cancer [11, 17]. While many colorectal cancers overexpress CEA, immunostaining and Western blots in the present study demonstrate a variability of expression of CEA in various colon cancer samples. The ability to bind to multiple CEACAMs may improve binding capacity of the antibody and enhance visualization of tumor margins and otherwise invisible.

Further potential uses of 6G5j-IR800CW include antibody-based targeted therapy for colon cancer. Prior studies have utilized a CEACAM5 antibody conjugated to a chemotherapy drug in colon cancer mouse models and demonstrated a reduction in tumor growth and increased survival [18]. The conjugation to the CEACAM antibody allowed for lower doses of the chemotherapy agent, which decreased toxicity associated with treatment [18]. In addition, Phase I/II clinical trials utilized anti-CEACAM5 antibodies conjugated to a chemotherapy drug for recurrent or metastatic colon cancer, which demonstrated safety and efficacy of the antibody-drug conjugate in this population [19]. Since the novel antibody utilized in the present study targets multiple CEACAM surface antigens, it may be useful for colorectal cancer therapy directed at any of the CEACAMs bound by 6G5j. A potential limitation of targeted therapy using this novel antibody is the binding capability of 6G5j to CEACAMs that are expressed on normal human cells, such as CEACAM1 expression on immune cells [4]. Future studies would help determine if the 6G5j antibody specifically binds any other normal human cells or tissue that expresses various CEACAMs.

Limitations of this study include the small sample size and short duration of time. Given the small sample size in this pilot study, there were not enough mice in each group to compare TLR between different PDOX models. Further studies comparing different PDOX models that demonstrate varying expression of CEACAMs can provide insight into the use of 6G5j-IR800CW in heterogenous colon cancer patient populations. Since the mice were euthanized and imaged shortly after administration of the antibody, we were unable to assess for long-term side effects. In future studies, long-term monitoring of mice after administration of 6G5j-IR800CW can provide information on possible long-term adverse reactions. In addition, future experiments will include immunohistochemistry of harvested intra-abdominal organs after the mice are euthanized, which can ascertain whether there is microscopic damage that is not grossly visible.

In addition, the Western blot staining of a human colon sample in the present study, as seen in Figure 3, demonstrated variable low expression of multiple CEACAMs, which may lead to background fluorescence of the surrounding colon. While this may be a limitation to the use of 6G5j-IR800CW in a clinical setting, the potential advantages of detecting multiple surface antigens and providing improved intra-operative visualization is promising. Future clinical studies would be useful to determine the effectiveness of this antibody-fluorophore conjugate in human patients with colorectal cancer.

Since fluorescence-guided surgery is a relatively novel therapeutic technique for oncologic disease, the application for various tumor types is still being studied [20]. While the technique described in the present study could be utilized for any type of colorectal cancer, fluorescence imaging would most likely be of particular use for cases of peritoneal carcinomatosis, rectal cancer or metastases otherwise invisible. Future studies are necessary to determine whether 6G5j-IR800CW would be useful in a clinical setting and to further delineate how fluorescence-guided surgery can be effective for colorectal cancer.

In conclusion, the anti-CEACAM1 antibody 6G5j may be a useful probe for primary colon cancer and metastasis detection for fluorescence-guided surgery. Since this mAb binds to multiple antigens that are commonly present in colon tumors, namely CEACAM1, 5 and 6, it may provide improved detection of cancer margins for tumors with variable expression of CEA and other CEACAMs. Further studies are necessary to validate the use of 6G5j-IR800CW in human patients with colorectal cancer.

## MATERIALS AND METHODS

### Antibody-fluorophore conjugation

6G5j mAb was obtained from B. B. Singer (Institute for Anatomy, Essen, Germany). IR800DyeCW NHS ester (LI-COR Biosciences Inc., Lincoln, NE) was conjugated to the antibody according to the manufacturer’s protocol. The combination was incubated under basic conditions at room temperature for 2 hours on a rotator plate (Fisher Scientific, Hampton, NH). Gel desalting columns were utilized for removal of unbound dye after conjugation (Thermo Fisher Scientific, Waltham, MA). The antibody-fluorophore mixture was added to the desalting columns and this was centrifuged three times at 1500 rpm for 3 minutes. The flow-through was then removed and stored in 4°C.

### Flow cytometry

Indicated CHO cell-line CEACAM transfectants and vector control cells (2 × 10^5^) were stained with mAb 6G5j (10 µg/ml) diluted in 3% FCS/PBS for 1 h on ice, washed twice with ice-cold PBS and incubated with FITC-conjugated goat anti-mouse F (ab´)2 (Jackson ImmunoResearch Cambridgeshire, UK) according to the manufacturer´s protocol. Background fluorescence was determined using isotype-matched IgG. To confirm CEACAM expression in the CEACAM4, CEACAM7, rat CEACAM1 and mouse CEACAM1 transfectants, respective control antibodies were applied. Stained samples were measured in a FACScalibur flow cytometer (BD Biosciences, Heidelberg, Germany) and the data were analyzed utilizing CellQuestPro software. Dead cells, identified by PI staining, were excluded from the measurement.

### Immunohistochemistry (IHC)

Sections of 4% paraffin-embedded normal human jejunum and colon were stained after demasking (10 min at 95°C in citrate buffer pH 6) with 10 µg/ml mAb 6G5j using HRP-coupled goat anti mouse IgG Fab_2_ antibody (Jackson ImmunoResearch Cambridgeshire, UK). Staining was developed with 3,3-diaminobenzidine (DAB, brown precipitate) with a hematoxylin counterstaining. Alternatively mAb 6G5j combined with the ZytoChem-Plus AP Polymer-Kit (Zytomed, Bargteheide, Germany) followed by DAPI staining was used according to the manufacturer´s protocol. DAB/hematoxylin stained samples and DAPI/fluorescent stained proteins were analyzed with a Leica DMI4000B microscope (20× objective, 10× eyepiece, total magnification 200×).

### Enzyme-linked immunosorbent assay (ELISA)

Indicated transfectants were lysed in RIPA buffer containing protease inhibitor cocktail set III (Calbiochem) and centrifuged at 18,000 rpm for 15 min at 4°C. The supernatants were diluted 1/10 with phosphate-buffered saline (PBS). Then, direct ELISA was performed with indicated CEACAM transfectant cell lysates dispensed at 100 µl per well in 96-well microplates (Nunc MaxiSorp, Nalge Nunc). After blocking with 350 µl 1% BSA/PBS, wells were incubated with 100 µl 5 µg/ml mAb 6G5j or 3 µg/ml rabbit panCEACAM pAb (LeukoCom, Essen, Germany) diluted in 0.5% BSA/PBS. Plates were washed three times with PBS and then incubated with HRP-coupled goat anti-mouse or anti-rabbit antibody (Jackson ImmunoResearch, Cambridgeshire, UK). Staining was developed with 100 µl/well tetramethylbenzidine solution (TMB X-tra, EcoTrak, BioTrend, Cologne, Germany) as the color developing reagent. Plates were read after 5–30 minutes of development and stopping with 200 mM H_2_SO_4_ solution in a microtiter plate reader (Sunrise Tecan, Maennedorf, Switzerland).

### Patient-derived tumor samples

Human patient colon cancers were resected from patients in the operating room at UCSD Thornton hospital under UCSD IRB protocol 140046 with informed patient consent. Per standard protocols with IRB approval and informed consent from the patient, these samples were used for establishment of PDOX models and preparation of tumor lysates. After dividing the tumor in the pathology lab, a portion of the tumor was placed in PBS on ice and transferred to the vivarium. Upon transfer to the vivarium, fragments of the tumor were implanted into the bilateral flanks of nude mice (*n* = 14). After intraperitoneal injection of ketamine, xylazine and acepromazine cocktail, the back of the mouse was prepped with 70% ethanol solution. A small incision was made over the back and four 1 mm tumor fragments were implanted on the bilateral shoulder and flank. The skin was closed with 6–0 nylon (Ethicon, Inc. NJ, USA). Subcutaneous patient-derived xenografts are maintained in our laboratory for ongoing studies.

### Colon cancer cell-line models

Human colon cancer cell-lines LS174T and HCT116 were obtained from the American Type Culture Collection (Manassas, VA). Nude mice were administered inhaled anesthetic with isoflurane and the flank was prepped with 70% ethanol solution. Under sterile conditions, LS174T cells (1 × 10^6^) reconstituted in 50 µL Matrigel Matrix (Corning Life Sciences, Corning, NY) and 50 µL cold PBS were injected into each flank of two nude mice. Two additional nude mice were used for injection of HCT116 cells (1 × 10^6^) reconstituted in Matrigel Matrix and PBS into each flank. Cell-line mouse models are maintained in our laboratory for ongoing studies.

### Western blotting

Western blotting of patient-derived tumor lysates and colon cancer cell-line lysates was performed once to identify CEACAM expression is various colon cancer samples. LS174T and HCT116 represent human colon cell-lines. Lung 3, C4, PM9, CM1, CM2, CM3, CM6, Liver 2, Liver 6, Lung 4, C14, Liver 5, PM12 and CM7 represent patient-derived colon cancer surgically obtained from our institution, described above. After surgical resection of tumor from patient-derived and cell-line subcutaneous xenografts described above, tumors were minced into small fragments. Cell lysis buffer, containing a cocktail of complete EDTA-free protease inhibitor cocktail tablet (Roche, Indianapolis, IN), RIPA buffer (Thermo Scientific, Rockford, IL) and phenylmethylsulfonyl fluoride (PMSF, Sigma, St. Louis, MO), was added to tumor fragments and placed on ice for two hours. Samples were centrifuged for 20 minutes at 12,000 rpm at 4°C. Supernatants were then collected and stored in –80°C. To determine protein concentration, samples were added to a 96-well plate and a cocktail of protein assay reagent A and protein assay reagent S (Bio-Rad Inc, USA) was added to each well. Protein assay reagent B (Bio-Rad Inc., USA) was then added to each sample. The 96-well plate was stored free from light for 15 minutes. Then, the protein concentration was analyzed using the Lowry Assay on a microplate reader (Bio-Rad Inc., USA).

Western blotting procedures were carried out at room temperature. A cocktail of 2× Laemmli Sample Buffer (Bio-Rad Inc., USA), 2-mercaptoethanol (Bio-Rad Inc., USA) and RIPA buffer was added to protein samples. Samples (50 μg/well) and dual plus molecular weight ladders (Bio-Rad Inc., USA) were separated by SDS-PAGE Gels with 12% gradient for approximately 120 minutes at 90V in running buffer (25 mM Tris base (Fisher BioReagents, Fair Lawn, NJ), 192 mM glycine, pH 8.3) (Bio-Rad Inc., USA). Proteins were transferred to Trans Blot turbo Mini-size nitrocellulose membranes with Transfer Buffer using the Bio-Rad Trans-Blot Turbo Transfer System for 30 minutes. Membranes were blocked with 5% non-fat milk in TBST (20 mM Tris, 150 mM NaCl, containing 0.05% Tween-20 (Sigma-Aldrich, St. Louis, MO), pH 7.4) for 60 minutes. The membrane was then incubated with conjugated 6G5j (0.83 mg/mL) at 4°C overnight. Removal of excess primary antibody was carried out by washing the membranes in TBST three times. The membrane was scanned with LI-COR Odyssey Infrared Imaging System (LI-COR, Lincoln, NE) and analysis was conducted using Image Studio Lite Version 5.2 software (LI-COR). Beta-actin was utilized as a control.

### Animals

Nude (nu/nu) mice, age 4–6 weeks (*n* = 11) purchased from Jackson Lab (Bar Harbor, ME), were used for this study. The animals were fed an autoclaved laboratory diet. All surgical procedures were performed with anesthesia by intraperitoneal injection of ketamine, xylazine and acepromazine cocktail reconstituted in phosphate-buffered saline (PBS). Mice were treated with buprenorphine for pain control after surgical procedures. At the time of intra-vital imaging (four weeks after tumor implantation), if tumor burden became too large as defined as tumor volume greater than 1000 mm^3^, or if mice displayed visible signs of distress and significant weight loss, mice were euthanized with CO_2_ inhalation, which was confirmed with cervical dislocation. All studies were approved by the San Diego Veterans Administration Medical Center Institutional Animal Care and Use Committee (IACUC, animal use protocol A17-020).

### Patient derived orthotopic xenograft (PDOX) models

Initial establishment of subcutaneous tumors: After intraperitoneal injection of 0.1 mL/20g mouse weight of a ketamine, xylazine and acepromazine cocktail, the back of the mouse was prepped with 70% ethanol solution. A small incision was made over the middle of the back through the skin and 1 mm^3^ tumor fragment (Lung 4) was implanted on the bilateral flank of the mouse. The tumor grew until the diameter was 5 mm. For orthotopic implantation, mice (*n* = 10) were administered intraperitoneal anesthesia cocktail as described above. The abdomen was prepped with 70% ethanol solution. An incision was made vertically in the midline of the abdomen through the skin and peritoneum. The cecum was carefully exposed and a 1 mm tumor fragment, previously grown subcutaneously, was implanted onto the serosa of the cecum using 8–0 surgical sutures (Ethicon Inc., NJ, USA). Implantation of C4, a patient-derived primary colon cancer, was performed on 5 mice and implantation of Lung4, a patient-derived colon cancer lung metastasis, was performed on 5 mice. The bowel was then returned to the peritoneal cavity, and the abdominal wall and skin were closed with 6-0 surgical sutures (Ethicon Inc., NJ, USA) [21–24].

### Mouse imaging

For non-invasive intra-vital imaging, the Pearl Trilogy Small Animal Fluorescence Imaging system was used (LI-COR, Lincoln. NE). The Pearl Trilogy is equipped for sensitive imaging at 700 and 800 nanometer near infrared fluorophores. Images were obtained with standard imaging settings, per the device company, and images were not altered prior to analysis. One mouse with bilateral flank subcutaneous patient-derived lung metastasis xenografts was utilized as a control mouse. Non-invasive imaging was performed after administration 25 μg of IR800 dye reconstituted in PBS was administered via tail vein injection. The mouse was then euthanized as described above and open imaging was performed to assess florescence of intra-abdominal organs.

Four weeks after orthotopic implantation, PDOX model mice were administered varying doses of 6G5j-IR800CW (25 μg or 50 μg) reconstituted in PBS via tail vein injection. Mice were then imaged 24 or 48 hours after administration to determine optimal antibody-fluorophore conjugate dose and optimal timing of imaging. Mice were euthanized as described above and laparotomy was performed for intra-vital imaging of the tumor and intra-abdominal organs. The intra-abdominal organs were inspected for evidence of gross toxicity.

Image analysis was performed with the Image Studio Software Small Animal Imaging Analysis Version 5.2 (LI-COR, Lincoln, NE) in order to determine the maximum fluorescence signal of the tumor and liver. Fluorescence signals were obtained by creating a background reference over the skin of the mouse. Fluorescence was quantified by automated areas, with a set standard deviation from background signal of 10 and minimum 250 pixels per area. Using these settings for all areas of interest, an automated area was defined for liver and tumor fluorescence. Maximum tumor fluorescence signal and maximum liver fluorescence signal were obtained from the software analysis. To determine tumor-to-liver ratio, maximum tumor signal was divided by maximum liver signal in each mouse. For statistical analysis, SPSS version 24 (IBM, Armonk, NY) was utilized. To determine optimal dosing of antibody-fluorophore conjugate, mean TLR was calculated at each time point, when the sample size was adequate for analysis.

## Abbreviations

CEA: Carcinoembryonic antigen
CEACAM: Carcinoembryonic antigen-related cell adhesion molecules
PDOX: Patient-derived Orthotopic Xenograft
MAb: Monoclonal antibody
TLR: Tumor-to-liver ratio
